# Cucumber seed polypeptides regulate RANKL-induced osteoclastogenesis through OPG/RANKL/RANK and NF-κB

**DOI:** 10.1007/s11626-023-00834-7

**Published:** 2023-12-20

**Authors:** Tao Yu, Xiao Liu, Meng Jiang, Yuanyue Li, Heng Su, Ben Niu

**Affiliations:** 1 Department of Gynecology, The First People’s Hospital of Yunnan Province, The Affiliated Hospital of Kunming University of Science and Technology, Kunming, 650032 Yunnan China; 2 Yunnan University of Traditional Chinese Medicine, Kunming, 650500 Yunnan China; 3 Department of Endocrinology and Metabolism, The First People’s Hospital of Yunnan Province, The Affiliated Hospital of Kunming University of Science and Technology, No. 157 Jinbi Road, KunmingYunnan, 650032 China

**Keywords:** Postmenopausal osteoporosis, Cucumber seed peptide, OPG/RANKL/RANK pathways, NF-κB pathways

## Abstract

Postmenopausal osteoporosis (PMOP) is a common disease that endangers the health of elderly women. Cucumber seeds have shown excellent therapeutic effects on PMOP, but the mechanism of cucumber seed peptide (CSP) remains unclear. The expression levels of NF-κB and osteoclast-related genes were detected by RT-qPCR. The levels of apoptosis-related proteins were detected by Western blotting. Nuclear translocation of NF-κB p65 and osteoclast formation were detected by immunofluorescence and tartrate-resistant acid phosphatase (TRAP) staining, respectively. ELISA was used to detect the expression levels of OPG, M-CSF, and RANKL. Hematoxylin–eosin (H&E) and TRAP staining were used to observe the effects of CSP on bone formation. In RAW264.7 cells, CSP (0.4 mg/L, 4 mg/L, and 40 mg/L) effectively inhibited the expression of osteoclast-related genes (Cathepsin-K, MT1-MMP, MMP-9, and TRAP). TRAP-positive multinucleated giant cells gradually decreased. Furthermore, NF-κB pathway activation downstream of RANK was inhibited. In bone marrow stromal cells (BMSCs), the expression levels of M-CSF and RANKL gradually decreased, and OPG gradually increased with increasing CSP concentrations. Treatment of RAW264.7 cells with pyrrolidine dithiocarbamate (PDTC, an inhibitor of NF-κB) prevented the formation of osteoclasts. Treatment with different concentrations of CSP effectively decreased the levels of RANKL and M-CSF in rat serum and increased the expression of OPG in the oophorectomy (OVX) rat model. Furthermore, different concentrations of CSP could ameliorate the loss of bone structure and inhibit the formation of osteoclasts in rats. CSP inhibits osteoclastogenesis by regulating the OPG/RANKL/RANK pathway and inhibiting the NF-kB pathway.

## Introduction

Postmenopausal osteoporosis (PMOP) is the most common type of osteoporosis caused by estrogen deficiency and typically occurs in postmenopausal women (Feng *et al*. 2021). Postmenopausal osteoporosis is mainly treated with bisphosphonates, hormone replacement therapy (HRT), and selective estrogen receptor modulators (SERMs), all of which are based on therapies that target the activity of bone-resorbing osteoclasts (Arceo-Mendoza and Camacho 2021; Brown 2021). However, these drugs can only reduce the sensitivity to fractures by approximately 50% (McNamara 2010). Therefore, new drugs without adverse side effects are needed for the treatment of osteoporosis.

Normal bone metabolism is managed bone destruction and bone formation, which maintain a dynamic balance. The osteoprotegerin (OPG)/receptor activator of nuclear factor NF-κB ligand (RANKL)/receptor activator of NF-κB (RANK) system is a signaling pathway that functions during bone reconstruction and osteoclast differentiation (Kovács *et al*. 2019). OPG is secreted by osteoblasts and is a RANKL bait receptor, which, as the only negative regulator of osteoclasts, can prevent the binding of osteoclasts to RANKL and block signal transduction, thus preventing bone resorption (Udagawa *et al*. 2021) (Lloyd *et al*. 2008). Studies have shown that after OPG knockdown, mice exhibit severe osteoporosis. RANKL belongs to the tumor necrosis factor ligand superfamily and is the only factor known to date that promotes the differentiation of osteoclast precursor cells into osteoclasts and enhances the activity of mature osteoclasts (McDonald *et al*. 2021). RANK is the only known receptor of RANKL, which is highly expressed on the surface of osteoclastic precursor cells and mature osteoclasts, directly promotes the differentiation and activation, and prevents osteoclast apoptosis. The binding of RANKL and RANK is critical for osteoclast survival, differentiation, and activation (Yasuda 2021). Therefore, the OPG/RANKL/RANK system is critical in bone remodeling and osteoclast differentiation.

The binding of RANKL produced by osteoblasts to RANK on osteoclasts can promote osteoclast differentiation and maturation (Dong *et al*. 2022). Certain drugs have been shown to alleviate PMOP through the NF-κB, AKT, and OPG/RANKL/RANK pathways. For example, fructose 1,6-diphosphate strontium and salmon calcitonin plus aspirin prevent bone loss in postmenopausal osteoporotic rats by regulating the OPG/RANKL/RANK pathway (Ma *et al*. 2012; Wei *et al*. 2015). Moreover, β-amyloid peptides promote bone formation by regulating OPG/RANKL/RANK and Wnt/β-catenin signaling (Yang *et al*. 2020). Dimethylbisphosphonate plus 99Tc-MDP can induce apoptosis in RAW264.7 cells by regulating the OPG/RANKL/RANK pathway, inhibiting RANK protein expression, and promoting MC3T3-E1 cell proliferation (Shen *et al*. 2019). Chen *et al*. (2020) reported that shikonin prevented osteoclastogenesis by inhibiting the downstream NF-κB signaling pathway via TRAF6 and RANK in osteoclasts in vitro. These findings have led to new ideas for the development and application of osteoporosis treatments.

Most researchers acknowledge the importance of nutrients in plant seeds for human health, and so the beneficial effects of various seeds, such as Anudeep seeds, on the host immune system have been examined (Anudeep *et al*. 2016). Cucumber seeds have a long history of providing remarkable results in folk treatments of diseases such as osteoporosis, osteoarthritis, and cervical spondylosis and have shown excellent therapeutic effects (Lin *et al*. 2019). Numerous studies have shown that peptides possess antioxidant, antihypertensive, antithrombotic, hypolipidemic, ACE inhibitory, immunoregulatory, and mineral absorption- or bioavailability-enhancing activities (Mohan *et al*. 2016). One study reported that casein phosphopeptides (CPPs) could promote the absorption of minerals in organisms (Rajapakse *et al*. 2005). Lin *et al*. (2019) showed that RANKL/M-CSF-induced osteoclast differentiation could be inhibited by deer cucurbit polypeptide (CCP) and simultaneously inhibit the activation of mid-downstream osteoclast-specific genes and transcription factors in RAW264.7 cells. Liquid fermentation of cucumber seeds by *Bacillus subtilis* showed that a specific cucumber seed peptide (CSP) was successfully produced (Wang *et al*. 2017). However, the mechanism of CSP in the host has not been reported.

However, whether the peptides (CSP) in cucumber seed powder can regulate postmenopausal osteoporosis through the OPG/RANK/RANKL and NF-KB pathways remains unclear. Consequently, we will continue to study the molecular and regulatory mechanisms of cucumber powder polypeptide in the prevention of postmenopausal osteoporosis.

## Materials and methods

### Osteoclast culture

RAW264.7 cells and BMSCs from the ATCC (Rockefeller, Maryland) cell bank were seeded in 10% fetal bovine serum and high glucose medium, placed at 37℃, and cultured under 5% CO_2_. During this time, the medium was changed every 2 d, and the cells were subcultured after 3–4 d. To induce osteoclast differentiation, cells within 15 passages were collected and seeded at a density of 2 × 10^4^ cells in 1 mL of medium. After the cells adhered to the plate for 24 h under the abovementioned culture conditions, the induction and differentiation medium was changed (basal medium + 50 ng/mL RANKL), and the cells were cultured for 6 d. CSP was obtained from Beyotime Science and Technology Co., Ltd. (Shanghai, China) and contained a mixture of several peptides (the molecular weights were approximately 3 ~ 12 kDa). Different concentrations of CSP (0.4 mg/L, 4 mg/L, and 40 mg/L) were cultured with RANKL-treated RAW264.7 cells or BMSCs.

### Reverse transcription-quantitative polymerase chain reaction (RT-qPCR)

We extracted total RNA from tissues and cells using a Total RNA Extractor Kit (Sangon Biotech, Shanghai, China). A cDNA synthesis kit (Vazyme, Nanjing, China) was used to reverse transcribe 2 μg of mRNA into cDNA, which was then diluted 10 times. One microliter of the prepared cDNA was used for qPCR. All primers (Table 1) used in this study were designed with Premier 5.0. The confidence of the PCR results was assessed based on the dissociation curve and cycle threshold (CT) values. The results were calculated by the 2^−ΔΔCt^ method after at least 3 repetitions. GAPDH was used to quantify the expression of various genes.

**Table 1.** Primer sequences

Name	Primer sequence
Cathepsin-K	Forward: 5′-CTTCCAATACGTGCAGCAGA-3′
	Reverse: 5′-TCTTCAGGGCTTTCTCGTTC-3′
TRAP	Forward: 5ʹ- GCTGGAAACCATGATCACCT-3ʹ
	Reverse: 5ʹ- GAGTTGCCACACAGCATCAC-3ʹ
MT1-MMP	Forward: 5′-CCCCGCTGCGGTGTTCCAGAC-3′
	Reverse: 5′-CTCCGCGGAGTCAAAGTGGGT-3′
MMP-9	Forward: 5′-CGTCGTGATCCCCACTTACT-3′
	Reverse: 5′-AACACACAGGGTTTGCCTTC-3′
M-CSF	Forward: 5′-GAACAGCCTGTCCCATCCATC-3′
	Reverse: 5′-TGAGGCCAGCTCAGTGCAA-3′
RANKL	Forward: 5′-CAGCATCGCTCTGTTCCTGTA-3′
	Reverse: 5′-CTGCGTTTTCATGGAGTCTCA-3′
OPG	Forward: 5′-ACCCAGAAACTGGTCATCAGC-3′
	Reverse: 5′-CTGCAATACACACACTCATCACT-3′
RANK	Forward: 5′-GGACGGTGTTGCAGCAGAT-3′
	Reverse: 5′-GCAGTCTGAGTTCCAGTGGTA-3′
NFATc1	Forward: 5′-TGGAGAAGCAGAGCACAGAC-3′
	Reverse: 5′-GCGGAAAGGTGGTATCTCAA-3′
c-Fos	Forward: 5′-CAAGCGGAGACAGATCAACTTG-3′
	Reverse: 5′-TTTCCTTCTCTTTCAGCAGATTGG-3′
GAPDH	Forward: 5′-AACTTTGGCATTGTGGAAGG-3′
	Reverse: 5′-ACACATTGGGGGTAGGAACA-3′

### Western blot analysis

In this study, proteins were extracted using a protein extraction kit (Sangon Biotech), and the bicinchoninic acid (BCA) assay (Sangon Biotech) was used to determine the total protein content. After denaturation for 5 min, total proteins were separated by 10% SDS-PAGE and transferred to polyvinylidene fluoride (PVDF) membranes (Millipore, Burlington, MA) via a constant current flow at 200 mA. Subsequently, the PVDF membranes were incubated with Bcl-2 (1:10,000), Bax (1:2000), Caspase-3 (1:5000), M-CSF (1:1000), RANKL (1:3000), OPG (1:3000), p-p65 (1:1000), p65 (1:1000), p-p38 (1:1000), p38 (1:5000), p-JNK (1:1000), JNK (1:1000), p-AKT (1:2000), AKT (1:2000), p-ERK (1:1000), ERK (1:1000), RANK (1:2000), NF-κB (1:1000), NFATc1 (1:10,000), and c-Fos (1:1000) antibodies (Abcam, Cambridge, MA) for 12 h at 4°C. TBS buffer was used to wash the PVDF membranes, and secondary antibodies (Abcam) were added and incubated at room temperature for 1 h. After the membranes were washed three times, chemiluminescent reagents were added, and the grayscale values of the bands were analyzed using ImageJ software. Each experiment was independently repeated 3 times. GAPDH was used to quantify the expression of various proteins.

### CCK-8 assay

We added 100 μL of the cell suspension (4 × 10^4^ cells/mL) to a 96-well plate, which was then placed in a 5% CO_2_ incubator at 37°C for 24 h. After the cells were transfected or treated, ten microliters of CCK-8 reagent was added and incubated for 2 h. Finally, an enzyme reader (ELX800, BioTeK, Winooski, VT) was used to measure the absorbance at 450 nm. Each experiment was independently repeated 3 times.

### 5-Ethynyl-2′-deoxyuridine (EdU) assay

The EdU reagent was diluted according to the instructions of the EdU kit (RIBOBIO, Guangzhou, China), after which 100 µL of 50 µM EdU reagent was added to the cells and incubated for 2 h. After the solution was discarded, the cells were washed with phosphate-buffered saline (PBS) and fixed with 4% paraformaldehyde solution for 30 min. After the paraformaldehyde solution was discarded, the cells were incubated with 2 mg/mL glycine solution on a decolorizing shaker for 5 min, washed with PBS, and incubated with 0.5% Triton X-100 penetrant on a shaker for 10 min. Following an additional wash with PBS, the cells were stained with the preprepared Apollo staining solution, destained, incubated in the dark on a shaker for 30 min, and washed with PBS. Next, nuclei were stained with 10 µL of 4′,6-diamidino-2-phenylindole (DAPI) in the dark for 30 min and washed with PBS, and image acquisition was performed under a fluorescence microscope.

### Cell apoptosis assay

Flow cytometry was used to measure apoptosis. After the different groups of cells were treated, the cells were collected in a flow tube, centrifuged at 4°C, washed with PBS, and resuspended. The cells were incubated according to the instructions for the Annexin V Combined Fluorescein Isothiocyanate/Propidium Iodide Kit (Abcam), and then Annexin V and PI fluorescence were detected at the referenced emission wavelengths using a flow cytometer (BD Biosciences) and FlowJo software.

### TRAP staining

For TRAP staining, slides containing cells were rinsed with PBS, fixed, and rinsed with deionized water at 25°C for 30 s. The cells were heated in a water bath to 37°C, immersed in staining solution, and incubated at 37°C for 1 h in the dark. They were then rinsed with deionized water, counterstained with hematoxylin for 90 s, rinsed with alkaline solution for several minutes, and observed under a microscope after being dried. For rat femur TRAP staining, after the paraffin sections were dewaxed, they were dipped in staining solution that was prewarmed to 37°C for 1 h, rinsed with distilled water, dehydrated with gradient alcohol, cleared with xylene, and sealed with neutral gum, and osteoclasts were observed under a microscope.

### Immunofluorescence analysis of NF-κB/p65 nuclear translocation

The NF-κB nuclear translocation assay was performed according to the instructions of the Nuclear Translocation Assay Kit (SN368, Beyotime). The cells were centrifuged to remove the culture medium, washed with PBS, and fixed with fixative for 5–15 min. After the fixative was removed, immunostaining blocking solution was added and incubated at 25°C for 1 h. The immunostaining blocking solution was aspirated, and the NF-κB p65 antibody was added and incubated for 1 h. After the primary antibody was removed, anti-rabbit Cy3 was added and incubated at room temperature for 1 h. After the samples were stained with DAPI for 15 min, images were acquired under a fluorescence microscope.

### Development of the oophorectomy (OVX) rat model

Six-month-old female SD rats were used to construct the model, and 5 groups were assigned: the sham operation group (sham), oophorectomy model group (OVX), CSP (Xiang Cao Biotechnology Ltd, Ningxia, China) low-dose treatment group (L-CSP), CSP medium-dose treatment group (M-CSP), and CSP high-dose treatment group (H-CSP). After 2 wk of adaptive feeding, a postmenopausal osteoporosis model was established according to the classic modeling method. In the sham group, 1% pentobarbital sodium (30 mg/kg body weight) was injected into the abdominal cavity, and anesthetized animals were fixed in the prone position. Under aseptic conditions, a dorsal median incision was performed to open the abdominal cavity near the lower abdomen, and the ovaries were identified. Fat tissue around the ovaries with the same weight as the bilateral ovaries was removed, and the incision was sutured in layers. In the OVX group, 1% pentobarbital sodium (30 mg/kg) was injected into the abdominal cavity, and the anesthetized animals were fixed in the prone position. Under aseptic conditions, a dorsal median incision was performed to open the abdominal cavity near the lower abdomen, the bilateral ovaries were identified and removed, and the incision was stitched in layers. In the L-CSP group, bilateral oophorectomy was performed as described in the model group, and 10 mg/kg CSP was administered orally every day beginning on the 7th day after surgery. In the M-CSP group, bilateral oophorectomy was performed as described in the model group, and 40 mg/kg CSP was administered orally every day beginning on the 7th day after surgery. In the H-CSP group, bilateral oophorectomy was performed as described in the model group, and 160 mg/kg CSP was administered orally every day beginning on the 7th day after surgery. Treatment was continued for 90 d. Rat femurs were collected for subsequent analysis.

### Enzyme-linked immunosorbent assay (ELISA)

Plates containing standard substances and serum samples from each group were incubated at 37℃ for 1 h and then washed with wash solution. Next, 50 µL of HRP-antibody solution was added to each cell well and incubated at 37℃ for 30 min and washed, followed by the addition of 50 µL substrate A and substrate B, incubation at 37℃ in the dark for 15 min, addition of 50 µL stop solution/well, and measurement of the OD value at a wavelength of 450 nm within 30 min.

### Hematoxylin–eosin (H&E) staining

Rat femurs were fixed for 48 h and then decalcified with 10% EDTA solution at room temperature for 6 wk. Femurs approximately 1 cm in length were dehydrated in a graded ethanol series (70%, 80%, 90%, 95%, 100% × 3, soaking in each solution for 60–70 min). Xylene (2 lanes, total 60 min) and immersion wax (3 lanes, total 70 min) were then applied. Paraffin-embedded sections were obtained using routine procedures. After being dewaxed with xylene, the sections were soaked in hematoxylin staining solution for 3 min, rinsed with tap water for 10 min, differentiated with 1% hydrochloric acid and ethanol for several seconds, rinsed again with running water for 10 min until the blue color was restored, stained with eosin staining solution for 3 min, and rinsed for 10 min. Transparent xylene and neutral resin mounts were observed under a microscope and photographed.

### Statistical analysis

The data are presented as the mean ± SD. For statistical comparisons, Student’s *t* test was used to compare two groups, and one-way ANOVA was performed for multiple groups. All cell experiments were performed with 3 parallel experiments, and each parallel experiment included 3 replicates. The animal experiment was performed in 5 parallel experiments.

## Results

### CSP promoted apoptosis in RAW264.7 cells

**Figure 1.**
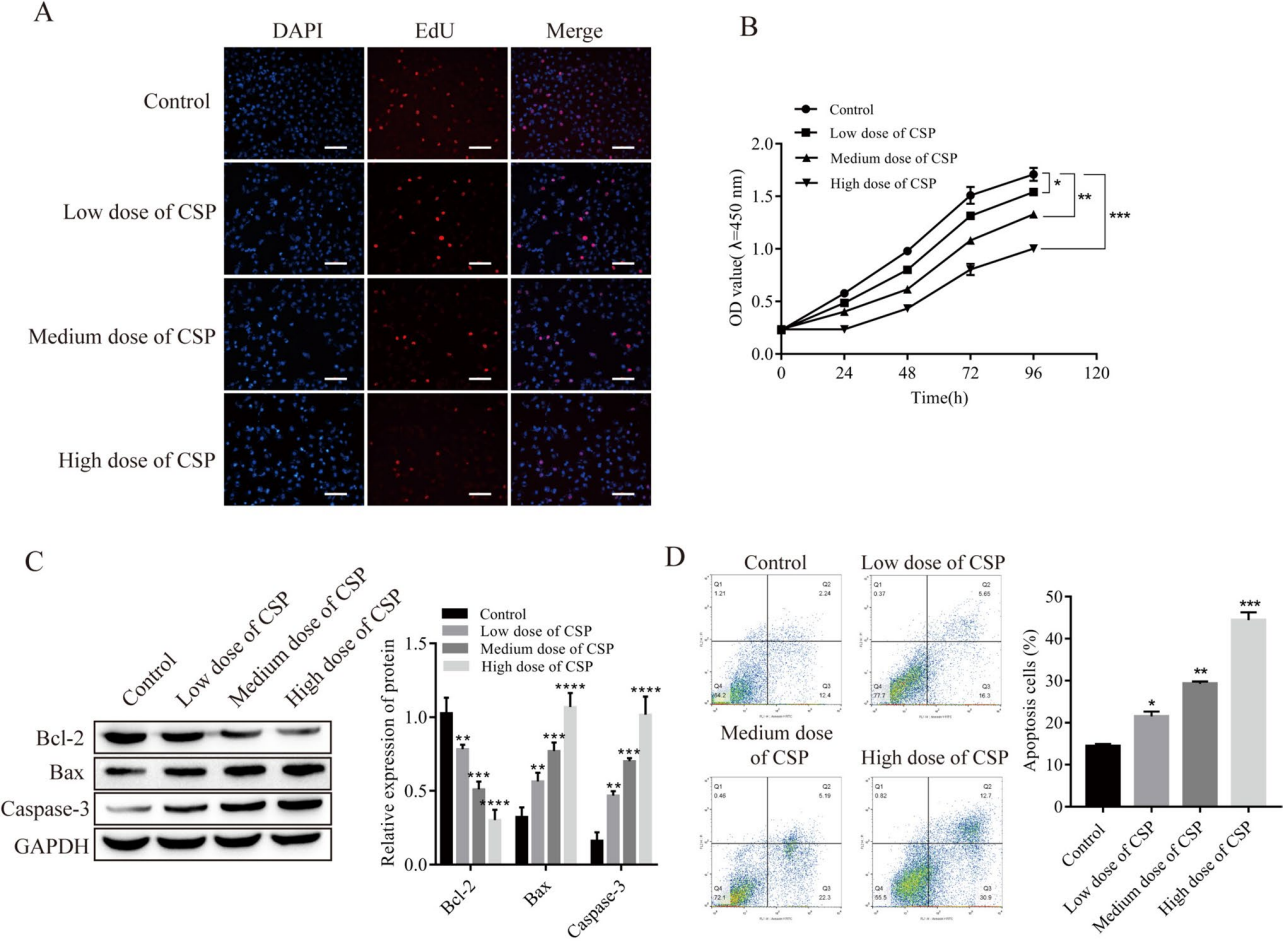
Effects of CSP on apoptosis in RAW264.7 cells (*n* = 3). (*A*) Cell proliferation was detected by EdU assays (*scale bar* = 100 μm). (*B*) Cell proliferation was detected by CCK-8 assays. (*C*) The expression of apoptosis-related proteins was detected by Western blot analysis. (*D*) Apoptosis was detected by flow cytometry. *P < 0.5, **P < 0.01, ***P < 0.001, ****P < 0.0001 vs. control. Cells were incubated with CSP for 48 h. Low, medium, and high doses of CPS refer to 0.4 mg/L, 4 mg/L, and 40 mg/L CPS, respectively.

In this study, different concentrations of CSP had an inhibitory effect on RAW264.7 cells (Fig. 1*A*, *B*). The protein expression of Bcl-2 in the CSP treatment group was downregulated, and the protein expression levels of Bax and Caspase-3 were upregulated; a more pronounced effect was observed in response to a higher concentration of CSP (Fig. 1*C*). Flow cytometry revealed that increasing CSP concentrations resulted in higher rates of apoptosis (Fig. 1*D*).

### CSP inhibits osteoclast formation in a dose-dependent manner

**Figure 2.**
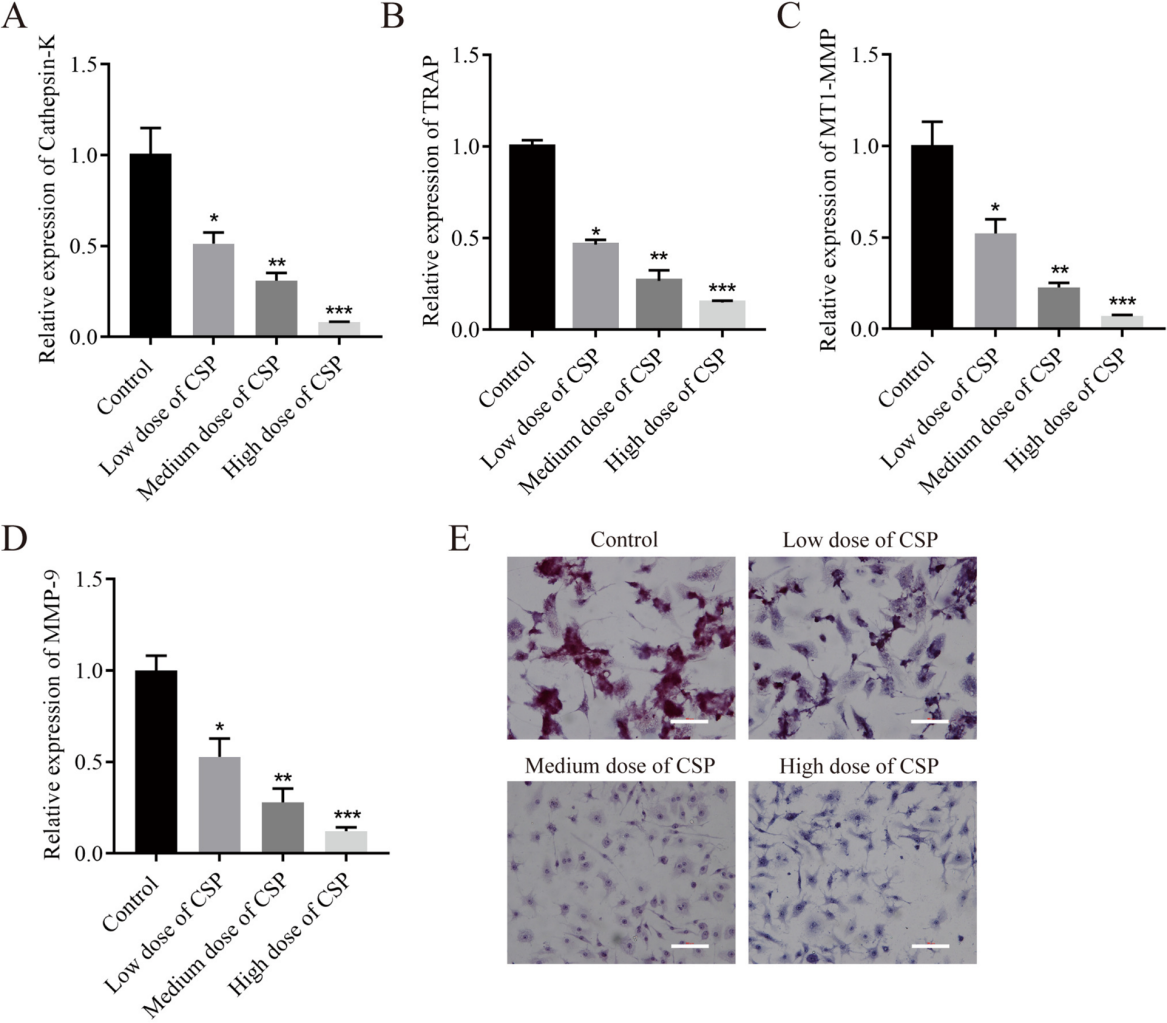
CSP inhibits osteoclast formation in a dose-dependent manner in RAW264.7 cells (*n* = 3). (*A*–*D*) The expression levels of Cathepsin-K, TRAP, MT1-MMP, and MMP-9 were detected by RT-qPCR. (*E*) TRAP staining was performed to determine the number of positive multinucleated giant cells (*scale bar* = 100 μm). *P < 0.5, **P < 0.01, ***P < 0.001 vs. control. Cells were incubated with CSP for 48 h. Low, medium, and high doses of CPS refer to 0.4 mg/L, 4 mg/L, and 40 mg/L CPS, respectively.

The effects of different concentrations of CSP on the formation of osteoclasts were observed. RT-qPCR showed that the expression levels of the osteoclast-related genes Cathepsin-K, TRAP, MT1-MMP, and MMP-9 were downregulated in the CSP-treated group in a dose-dependent manner (Fig. 2*A*–*D*). Based on the TRAP staining results, the number of TRAP-positive multinucleated giant cells in the CSP group decreased with increasing CSP concentrations (Fig. 2*E*).

### CSP regulates the OPG/RANKL/RANK pathway

**Figure 3.**
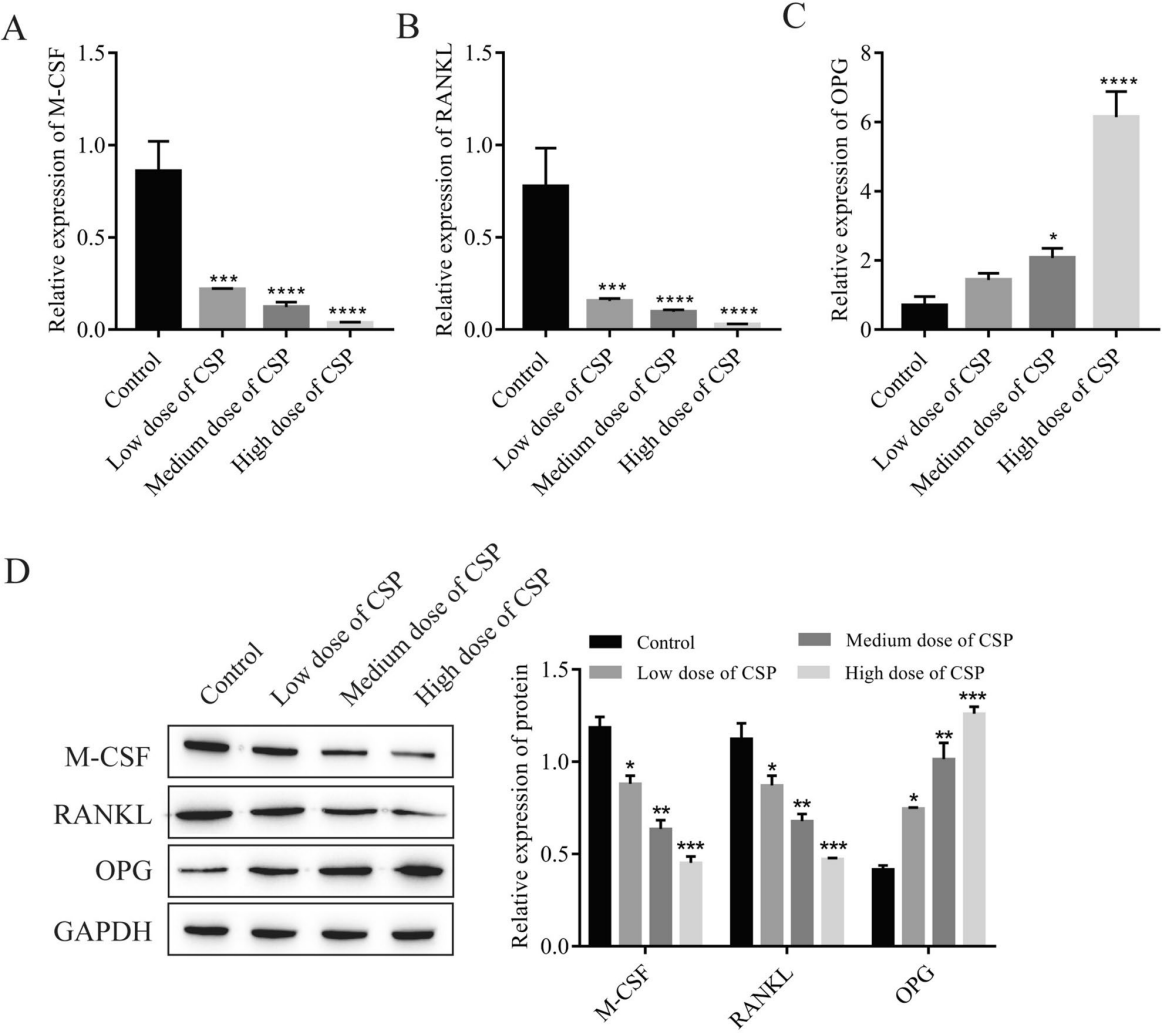
CSP regulates the OPG/RANKL/RANK signaling pathway in BMSCs (*n* = 3). (*A*–*C*) The expression of M-CSF, RANKL, and OPG was detected by RT-qPCR. (*D*) The protein expression of M-CSF, RANKL, and OPG was detected by Western blot analysis. *P < 0.5, **P < 0.01, ***P < 0.001, ****P < 0.0001 vs. control. Cells were incubated with CSP for 48 h. Low, medium, and high doses of CPS refer to 0.4 mg/L, 4 mg/L, and 40 mg/L CPS, respectively.

The levels of OPG/RANKL/RANK pathway–related proteins in BMSCs treated with different concentrations of CSP were detected. The expression levels of M-CSF and RANKL gradually decreased, and the levels of OPG gradually increased with increasing CSP concentrations (Fig. 3*A*–*C*). The western blot results were consistent with the RT-qPCR results (Fig. 3*D*).

### CSP inhibits the activation of NF-κB by inhibiting the RANKL-RANK signaling pathway

**Figure 4.**
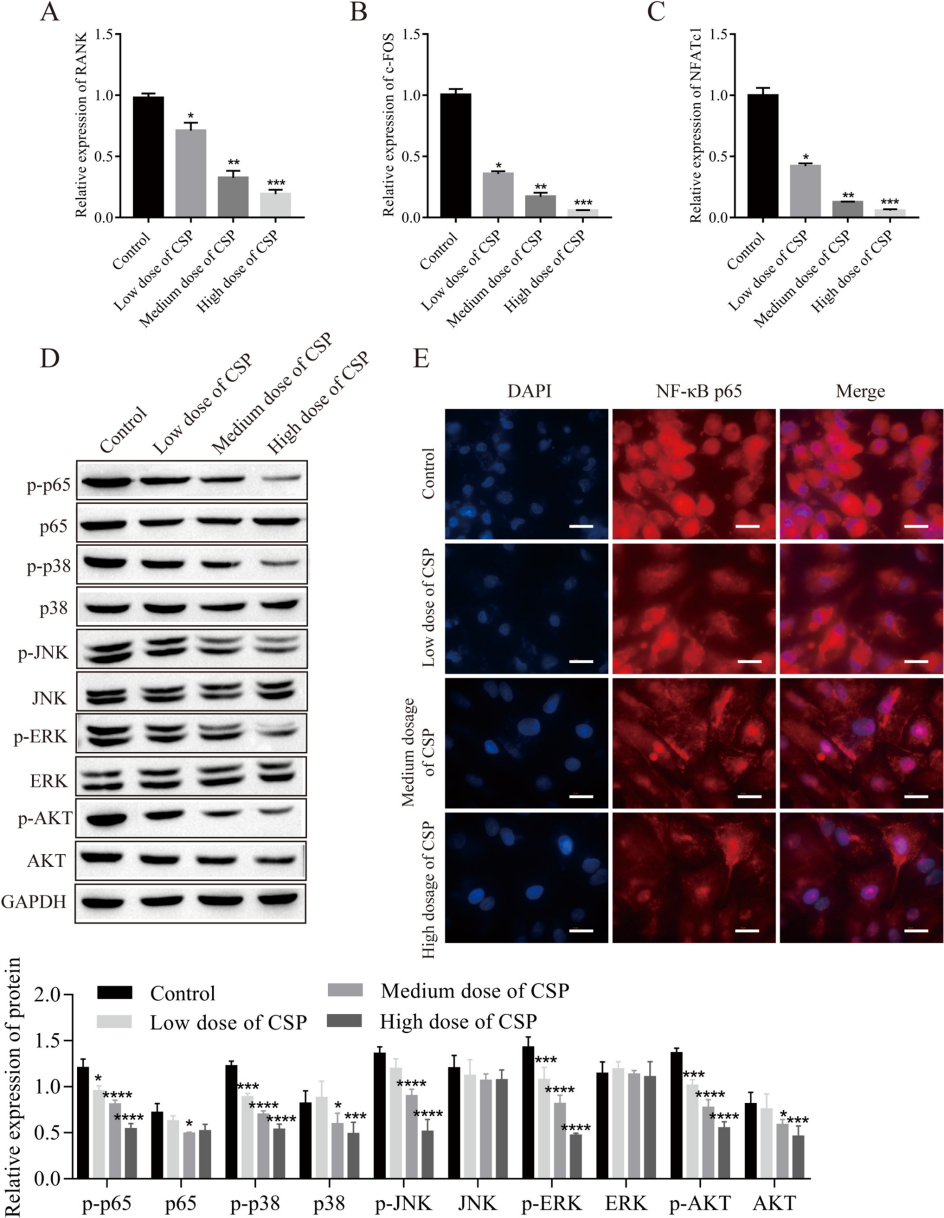
CSP inhibits the activation of NF-κB by inhibiting the RANKL-RANK signaling pathway in RAW264.7 cells (*n* = 3). (*A*–*C*) The expression of RANK, NFATc1, and c-FOS was detected by RT-qPCR. (*D*) Protein expression was detected by Western blot analysis. (*E*) Nuclear translocation of NF-κB p65 was detected by immunofluorescence analysis (*scale bar* = 20 μm). *P < 0.5, **P < 0.01, ***P < 0.001 vs. control. Cells were incubated with CSP for 48 h. Low, medium, and high doses of CPS refer to 0.4 mg/L, 4 mg/L, and 40 mg/L CPS, respectively.

The effects of CSP on the NF-κB pathway were examined and showed a gradual decrease in RANK, NFATc1, and c-Fos expression with increasing CSP concentrations (Fig. 4*A*–*C*). The phosphorylation levels of p65, p38, ERK, JNK, and AKT were decreased after CSP treatment (Fig. 4*D*). Concurrently, an immunofluorescence assay was used to examine the nuclear translocation of NF-κB p65, and the expression of p65 in the nucleus decreased with increasing CSP concentrations (Fig. 4*E*).

### CSP enhances the inhibitory effect of PDTC on osteoclast formation and downstream signaling pathway activation

**Figure 5.**
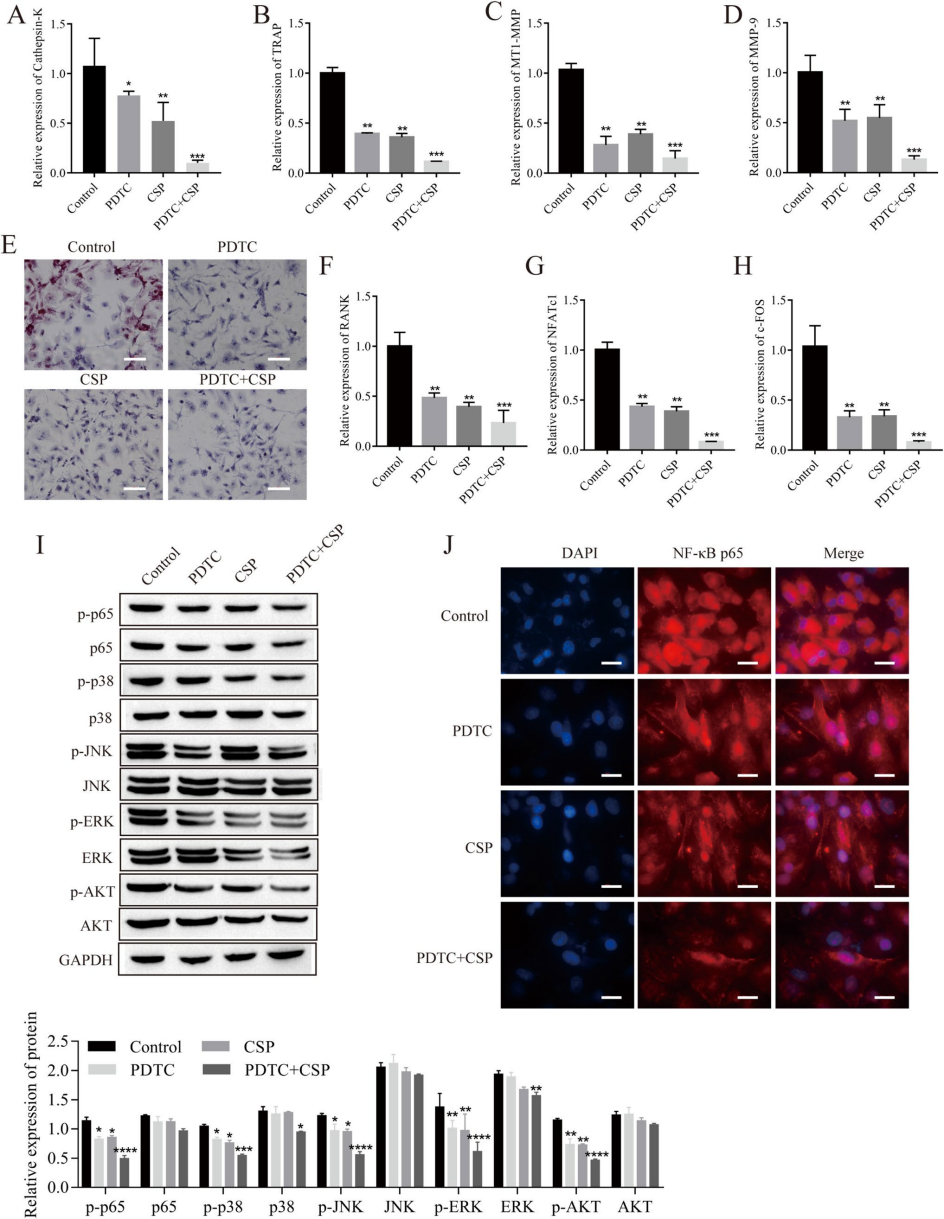
CSP enhances the inhibitory effect of PDTC on osteoclast formation and downstream signaling pathway activation in RAW264.7 cells (*n* = 3). (*A*–*D*) The expression levels of Cathepsin-K, TRAP, MT1-MMP, and MMP-9 were detected by RT-qPCR. (*E*) The number of positive multinucleated giant cells was detected by TRAP staining (scale bar=100 μm). (*F*–*H*) The expression of RANK, NFATc1, and c-FOS was detected by RT-qPCR. (*I*) Protein expression was detected by Western blot analysis. (*J*) Nuclear translocation of NF-κB p65 was detected by immunofluorescence analysis (*scale bar* = 20 μm). *P < 0.5, **P < 0.01, ***P < 0.001, ****P < 0.0001 vs. control. Cells were incubated with CSP (40 mg/L) or PDTC (4 μmol/L) for 48 h.

Cells were treated with PDTC, an inhibitor of the NF-κB signaling pathway, which inhibited osteoclast formation, and CSP enhanced the inhibitory effect of PDTC on osteoclast formation. The expression of the osteoclast-related genes Cathepsin-K, TRAP, MT1-MMP, and MMP-9 was downregulated in the PDTC group. The same effect was observed in the PDTC + CSP group, and the degree of downregulation was greater than that in the PDTC group (Fig. 5*A*–*D*). Furthermore, there were fewer TRAP-positive multinucleated giant cells in the PDTC group than in the control group, and there were fewer TRAP-positive multinucleated giant cells in the PDTC + CSP group than the PDTC group (Fig. 5*E*). In addition, PDTC inhibited the activation of downstream signaling pathways, and this effect was enhanced by CSP. In the PDTC group, the RT-qPCR results showed downregulated expression of RANK, NFATc1, and C-FOS, and the degree of the downregulation was increased in the PDTC + CSP group (Fig. 5*F*–*H*). The phosphorylation levels of p65, P38, ERK, JNK, and AKT were downregulated, and the degree of the downregulation was greater in the PDTC + CSP group (Fig. 5*I*). The immunofluorescence results revealed a reduction in nuclear expression of NF-κB p65 in the PDTC group and even lower nuclear expression in the PDTC + CSP group (Fig. 5*J*).

### CSP ameliorates bone structure loss in rats

**Figure 6.**
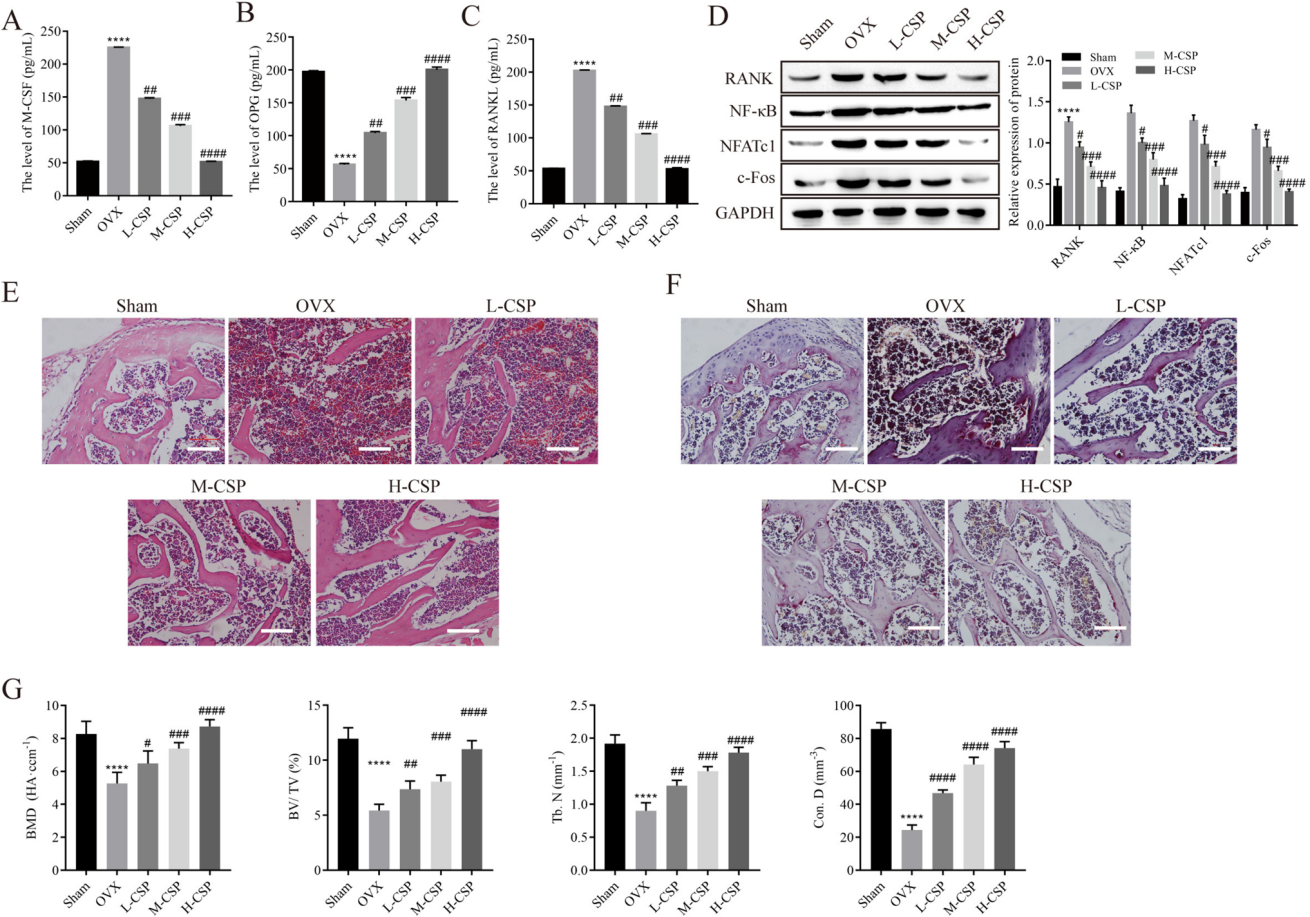
CSP ameliorates bone structure loss in rats (*n* = 5). (*A*–*C*) The expression levels of M-CSF, OPG, and RANKL were detected by ELISA. (*D*) Protein expression was detected by Western blot analysis. (*E*) The effect of CSP on bone structure observed by H&E staining (*scale bar* = 100 μm). (*F*) The effect of CSP on osteoclast formation observed by TRAP staining (*scale bar* = 100 μm). (*G*) Quantitative analysis of BMD, BV/TV, Tb. N, and Conn. D in each group of rats. ****P < 0.0001 vs. control; ^##^*P* < 0.01, ^###^*P* < 0.001, ^####^*P* < 0.0001 vs. OVX. Low, medium, and high doses of CPS refer to 10 mg/kg/d, 40 mg/kg/d, and 100 mg/kg/d CPS, respectively. BMD, bone mineral density; BV/TV, bone volume/total volume; Tb. N, trabecular number; Conn. D, connectivity density.

Serum levels of OPG, RANKL, and M-CSF in rats were detected by ELISA. The results showed that compared with that in the sham group, the expression of OPG was downregulated and the expression of RANKL and M-CSF was upregulated in the OVX group. However, after treatment with different concentrations of CSP, the expression of OPG increased, while the expression of RANKL and M-CSF was inhibited, and this effect was strongest in the H-CSP group (Fig. 6*A*–*C*). Compared with those in the sham group, the expression levels of RANK, NF-κB, NFATc1, and c-Fos were upregulated in the OVX group; these expression levels were downregulated in the CSP group; and the degree of downregulation was greatest in the H-CSP group (Fig. 6*D*). H&E staining showed that the trabecular bones of rats in the sham group had uniform thickness, complete structures, and small spaces. Rats in the OVX group showed numerous changes, such as a larger bone marrow cavity, thinner trabecular bone, and enlarged space, and the overall structure was severely altered. Treatment with CSP improved the lack of bone structure in rats. In the H-CSP group, the boundary between cortical bone and bone marrow was clear, and the structure was complete and almost normal (Fig. 6*E*). In the OVX group, TRAP staining revealed an increase in irregularly located wine-red osteoclasts at the edge of the trabecular bone, and osteoclasts dose-dependently decreased in the L-CSP, M-CSP, and H-CSP groups (Fig. 6*F*). Compared with those in the sham group, the bone mineral density (BMD), bone volume fraction (bone volume/total volume, BV/TV), trabecular number (Tb.N), and connectivity density (Conn. D) in the OVX group were significantly decreased, indicating successful modeling. BMD, BV/TV, Tb.N and Conn. D were significantly increased by the administration of CSP, and the therapeutic effect in the high-dose group was significantly better than that in the low- and medium-dose groups (Fig. 6*G*). These results suggest that CSP can protect against bone microstructural damage induced by OVX.

## Discussion

In postmenopausal women, bone resorption is greater than bone formation due to declines in ovarian function and estrogen levels, resulting in systemic osteopenia and destruction of the bone microstructure (McClung *et al*. 2017). In addition, an important factor in osteopenia is the imbalance between osteoclast-driven bone resorption and osteoblast-driven bone formation (Dimitriou *et al*. 2011; Cui *et al*. 2019). Studies have shown that the active ingredients in natural traditional Chinese medicines can reduce degradation of the bone microstructure to maintain the balance of bone resorption (An *et al*. 2016). Thus, studies of the related mechanisms of these components for osteoporosis drug treatment are meaningful.

Previous research has demonstrated that natural compounds have great potential in the treatment of osteoporosis (Tao *et al*. 2018). For example, RANKL-induced osteoclast differentiation and maturation can be inhibited by hesperetin (Hes) from citrus fruits, which has protective effects on osteoporotic animals (Zhang *et al*. 2018). As a raw material for separating peptides, cucumber seeds are widely used in the field of modern Chinese medicine and can supplement calcium, strengthen bones, and treat osteoporosis. This study showed that CSP inhibited RANKL-induced osteoclastogenesis in a concentration-dependent manner. Moreover, bone remodeling requires intimate cross-talk between osteoclasts and osteoblasts (Kular *et al*. 2012), and bone homeostasis depends on the resorption of bone by osteoclasts and the formation of bone by osteoblasts (Chen *et al*. 2018). In this study, CSP inhibited osteoclast differentiation and maintained bone homeostasis by regulating the expression of M-CSF, RANKL, and OPG in the OVX model, and CSP ameliorated bone structure loss in OVX rats. This study confirmed that the cucumber seed extract CSP alleviated the symptoms of osteoporosis in rats by mediating osteoclastic and osteoblastic bone differentiation in vivo*.*

OPG/RANL/RANK is an important signaling pathway that regulates the differentiation and induction of osteoclasts, which can directly or indirectly affect senile and postmenopausal osteoporosis (Udagawa *et al*. 2021; Zhao *et al*. 2020). OPG and RANK are receptors of RANKL. On the surface of osteoclasts, the binding of RANKL to RANK stimulates the activation of osteoclasts, while OPG can block binding of RANKL to RANK to inhibit bone resorption (Maria *et al*. 2018; Yasuda *et al*. 1998). M-CSF is also a key factor in osteoclast formation, and the M-CSF signaling pathway induces osteoclast proliferation and maintains osteoclast survival. In our study, CSP inhibited the expression of M-CSF and RANKL and promoted the expression of OPG by regulating the OPG/RANKL/RANK pathway while inhibiting the osteoclast-associated genes MT1-MMP, TRAP, MMP-9, and Cathepsin-K, which have been reported to be markers associated with osteoclast differentiation and function (Junrui *et al*. 2016).

After RANKL binds to RANK, downstream signaling pathways are activated, mainly through RANK-induced ubiquitination of the junction molecule TRAF6, which is involved in osteoclast differentiation and activation of the NF-κB, PI3K/Akt, and MAPK (P38, ERK, JNK) pathways (Boyle *et al*. 2003; He *et al*. 2014). Activation of NF-κB signaling results in the nuclear translocation and phosphorylation of NF-κB p65, which contributes to osteoclast maturation and enhances bone resorption activity (Ding *et al*. 2022). In addition, the NF-κB pathway has multiple regulatory effects on osteoclastogenesis, and abnormal activation of NF-κB signaling in osteoclasts is related to osteoclast activity. For instance, NF-κB modulators such as parthenolide and NEMO-binding domain peptide exert therapeutic effects on inflammation-induced bone destruction in mouse models (Xu *et al*. 2009). In the present study, we found that CSP significantly reduced the protein expression of AKT, P65, ERK, P38, and JNK; inhibited downstream signaling pathway activation; and inhibited nuclear NF-κB p65 translocation. These results indicate that abnormal activation of the NF-κB signaling pathway in osteoclasts is closely related to the activity of osteoclasts, and the inhibitory effect of CSP on the NF-κB pathway in osteoclasts is critical for the treatment of postmenopausal osteoporosis. In addition, CSP enhanced the expression of osteoclast effector genes, which are functional molecules in osteoclast development. Among these osteoclast effector genes, NFATC-1 is considered to be an important regulator of enhanced osteoclast formation (Boyce *et al*. 2015). In addition, defective expression of c-Fos, a key transcription factor that affects osteoclast differentiation, in mice leads to severe osteosclerosis due to complete blockade of osteoclast differentiation (Matsuo *et al*. 2000; Wagner and Eferl 2005). In this study, we found that with increasing CSP concentrations, the expression of NFATc1 and c-Fos in RANKL-induced RAW264.7 cells gradually decreased.

## Conclusion

Overall, the current findings show that CSP can inhibit RANKL-induced osteoclast formation and ameliorate bone structure loss in rats. These effects are mainly achieved through regulating the OPG/RANKL/RANK pathway and inhibiting the NF-kB pathway.. Therefore, CSP is a potential treatment for postmenopausal osteoporosis, although its clinical application requires further research.

## Data Availability

The datasets used and/or analyzed in the current study are available from the corresponding author upon reasonable request.
